# The Association Between Believing in Free Will and Subjective Well-Being Is Confounded by a Sense of Personal Control

**DOI:** 10.3389/fpsyg.2018.00623

**Published:** 2018-05-07

**Authors:** Peter L. T. Gooding, Mitchell J. Callan, Gethin Hughes

**Affiliations:** Department of Psychology, University of Essex, Essex, United Kingdom

**Keywords:** free will, choice, control, satisfaction with life, subjective well-being, perceived stress, depression

## Abstract

The extent to which an individual believes in free will is associated with a number of positive life outcomes, including their own subjective well-being. However, it is not known whether the belief that one has free will *per se* is uniquely associated with subjective well-being over and above potential confounding variables. We examined a sense of personal control as one such confound—specifically, whether the association between free will belief (FWB) and subjective well-being is based, in part, on the degree to which an individual feels a sense of personal control over their life. In Study, 1 trait-level belief in personal control significantly uniquely predicted satisfaction with life and stress, over and above the contribution of FWB. In Study 2, within-person daily fluctuations in stress and depression were not significantly predicted by daily changes in FWB over and above the contribution of personal control/choice. The findings provide new insight into the relationship between FWB and subjective well-being.

## Introduction

A growing body of evidence has shown that believing in free will is associated with a variety of positive life outcomes, including feeling grateful for past events (MacKenzie et al., 2014), better job performance (Stillman et al., 2010), higher academic achievement (Feldman et al., 2016), passionate love (Boudesseul et al., 2016), satisfaction with life (Li et al., 2017), and lower levels of perceived stress (Crescioni et al., 2015).

Nonetheless, the extent to which belief in free will *per se* is associated with positive life outcomes or whether some third variable is driving these associations remains to be explored. One possibility is that the relationship between free will beliefs (FWBs) and positive life outcomes, such as satisfaction with one’s life, might be confounded by a sense of personal control. Indeed, it is well-established that a sense of personal control is positively associated with many of the same positive life outcomes that relate to FWB, including subjective well-being (for reviews, see Myers and Diener, 1995; Peterson, 1999; Ross and Mirowsky, 2013). Thus, it is unclear whether FWB are uniquely associated with indicators of subjective well-being over and above a sense of personal control.

In their work exploring lay understandings of free will, Monroe and Malle (2010, 2014) found that people’s definitions of what it means to have free will differed from philosophical understandings that typically view free will as the ability for our conscious minds (or a soul) to make decisions, regardless of brain states or prior causal events (Harris, 2012). Rather, people defined free will as their freedom to make choices and the ability to act without constraints—that is, their sense of personal control (see also Baumeister and Monroe, 2014). Thus, insofar that our participants’ lay concepts of FWB are specifically tied to having a sense of personal control, then individual differences in a sense of personal control might better predict subjective well-being than individual differences in FWB. Consistent with this idea, Monroe et al. (2017) found that people’s beliefs that an agent who committed an immoral act had the capacity to choose their actions better predicted judgments of their blameworthiness than did their beliefs that the agent had free will. We reasoned that the known association between FWB and subjective well-being might be confounded by a sense of personal control.

Across two studies, we compared the relative predictive utility of perceived control/choice and FWB across various indicators of subjective well-being. Study 1 investigated the degree to which personal control and FWB uniquely predicted satisfaction with life and perceived stress. Study 2 assessed how daily changes in perceived choice/control and FWB predicted life stress and depression across a 2-week period. Given the foregoing analysis, we predicted that the known associations between FWB and subjective well-being could be explained, in part, by people’s perceived ability to have choice and to control their lives. In Study 2 we also assessed participants’ qualitative definitions of free will, to investigate whether they fit with previously reported lay conceptions of FWB (cf. Monroe and Malle, 2010).

## Study 1

### Method

#### Participants

Participants from the United States were recruited through Amazon’s Mechanical Turk (*N* = 284). Demographic information was not collected (but see Levay et al., 2016, for information on the typical demographic composition of Mechanical Turk workers). Nineteen additional participants were excluded because of duplicate IP addresses (*n* = 6) or failing a basic attention check item (*n* = 13). A power analysis showed that our sample size had 80% power to detect “small-to-medium” effect sizes (*f*^2^ = 0.028; α = 0.05, two-tailed) in our multiple regression analysis.

#### Procedure and Materials

Participants were instructed that they would complete a survey about their beliefs and opinions. We measured participants’ belief in free will using a single-item, graphical slider scale (“Using the slider provided, please indicate the extent to which you believe in free will”). The scale ranged from 0 (*no belief in free will*) to 100 (*absolute belief in free will*), and the starting position of the slider was set to the mid-point of the scale. This measure has been shown to have good convergent (Schooler et al., 2014) and predictive (e.g., Feldman et al., 2016) validity, and single-item free will measures have been shown to be sensitive to experimental manipulations of FWBs (MacKenzie et al., 2014; Nahmias et al., 2014; Monroe et al., 2017).

Participants’ sense of personal control was gauged using a five-item measure (e.g., “Other people determine most of what I can and cannot do”; “There is little I can do to change many of the important things in my life”; “I can do just about anything I really set my mind to”; Chou et al., 2016, adapted from Lachman and Weaver, 1998). Participants indicated their level of agreement on a five-point scale ranging from 1 (*strongly disagree*) to 5 (*strongly agree*). Higher scores indicate a greater sense of personal control.

Participants’ perceived stress was measured using two items: “In the past year, how would you rate the amount of stress in your life (at home and at work)?” (1 = *no stress* to 6 = *extreme stress*; Littman et al., 2006) and “Stress means a situation in which a person feels tense, restless, nervous, or anxious or is unable to sleep at night because his/her mind is troubled all the time. Do you feel this kind of stress these days?” (1 = *not at all* to 6 = *very much*; Elo et al., 2003). Responses to the two items were highly correlated (*r* = 0.73, *p* < 0.001) and therefore averaged to form a composite measure of perceived stress.

Participants’ life satisfaction was measured using Diener et al. (1985) widely used Satisfaction With Life Scale (SWLS), which is comprised of five items (e.g., “In most ways my life is close to my ideal”; 1 = *strongly disagree* to 7 = *strongly agree*). Alpha reliabilities for all measures with more than one item are shown in **Table 1**.

**Table 1:** Descriptive statistics and correlations among measures in Study 1.

Measures	Mean (*SD*)	1	2	3	4
(1) FWB	82.52 (19.57)	–			
(2) Control	3.82 (0.83)	0.426^∗∗^	(0.83)		
(3) SWLS	4.20 (1.44)	0.254^∗∗^	0.510^∗∗^	(0.97)	
(4) Stress	3.61 (1.25)	-0.145^∗^	-0.424^∗∗^	-0.409^∗∗^	(0.83)

*SWLS, the Satisfaction With Life Scale. When applicable, alpha reliabilities are presented in parentheses along the diagonal. ^∗^p < 0.05; ^∗∗^p < 0.01.*

### Results and Discussion

**Table 1** presents descriptive statistics, alpha reliabilities, and correlations among the measures. All of the measures correlated significantly in the expected directions. FWB positively correlated with sense of personal control, and both correlated positively with SWL and negatively with perceived stress.

A multiple regression analysis showed that sense of personal control, *b* = 0.85, β = 0.49, *SE* = 0.098, *t*(281) = 8.65, *p* < 0.001, *sr*^2^ = 0.20, but not FWB, *b* = 0.003, β = 0.05, *SE* = 0.004, *t*(281) = 0.81, *p* = 0.42, *sr*^2^ = 0.002, uniquely predicted scores on the SWLS. Likewise, personal control, *b* = -0.67, β = -0.443, *SE* = 0.090, *t*(281) = -7.42, *p* < 0.001, *sr*^2^ = 0.20, but not FWB, *b* = 0.003, β = 0.04, *SE* = 0.004, *t*(281) = 0.73, *p* = 0.46, *sr*^2^ = 0.002, uniquely predicted perceived stress.

Because confounding relationships are a special case of indirect relationships (MacKinnon et al., 2000), we tested whether there was a significant decrease in the regression weight for FWB when modeled with a sense of personal control to predict each of SWL and perceived stress compared to when FWB was modeled alone. Using Preacher and Hayes’s (2008) bootstrapping procedure for testing indirect effects (10,000 resamples for each analysis), the relationships between FWB and SWL (indirect relationship = 0.015, β = 0.21, 95% bias-corrected and accelerated confidence interval: 0.011, 0.022) and FWB and perceived stress (indirect relationship = -0.012, β = -0.19, 95% bias-corrected and accelerated confidence interval: -0.017, -0.008) were significantly reduced from their zero-order validities when statistically controlling for a sense of personal control.

These results suggest that the associations between FWB and SWL and FWB and perceived stress are largely due to co-variation between FWB and a sense of personal control. One limitation, however, is that our multiple regression approach fails to take measurement unreliability into account, which Westfall and Yarkoni (2016) showed can lead to spurious conclusions when testing for incremental validity. To address this issue, we replicated our results using structural equation modeling. Specifically, using the *lavaan* package in R (Rosseel, 2012), we specified measurement models for each predictor (sense of personal control and FWB) and each outcome to predict the latent outcome variables (separately for SWL and perceived stress) from the latent predictor variables. Because FWB was measured by a single indicator and therefore reliability could not be estimated empirically, we had to constrain the reliability of the FWB slider scores in our models. Following Westfall and Yarkoni’s (2016) recommendation, we tested a range of assumed reliabilities for our FWB measure (from 0.2 to 1 in increments of 0.2). Assuming the reliability for the FWB scores was as low as 0.4 (models failed to converge when the reliability of the FWB scores was assumed to be 0.2), analyses showed that a sense of personal control significantly predicted both SWL, *Z* = 3.99, *p* < 0.001, and perceived stress, *Z* = -3.88, *p* < 0.001, over and above FWB. In no case did FWB significantly predict the outcome variables over and above the contributions of sense of personal control (all *p*s > 0.13; the partial relationship between FWB and stress tended to become more *positive* when we assumed lower reliability for FWB scores).

## Study 2

### Method

#### Participants

The final sample of participants were 88 staff or students from the University of Essex (*M*_age_ = 24.18, *SD*_age_ = 6.50; 77% female) who participated in exchange for a monetary reward ($1 for an initial session and $1 for every daily diary completed) and the chance to win gift cards. Two additional participants completed measures during an initial session but did not complete any of our focal daily measures. The final sample size was determined by how many participants we could recruit within our monetary budget and time constraints.

#### Procedure and Measures

Participants attended an initial laboratory session where they completed a variety of measures unrelated to the current project. Of relevance here, during this initial session participants were asked to respond to an open-ended question about their FWBs: “Please explain what you think it means to have free will” (Monroe and Malle, 2010). Responses to this question were coded by two raters using Monroe and Malle (2010) original coding scheme. We included this question to replicate Monroe and Malle’s (2010) findings surrounding what “free will” means to people.

At the end of the initial session, participants were informed that they would receive daily emails including a link to a 10-min survey. The daily surveys were emailed to participants every day for 14 days at 5:00 PM; they had until 3:00 AM to complete the daily surveys. Participants who failed to complete more than five daily surveys were removed from the study (i.e., no longer sent the email links), but all data from participants who completed at least one daily survey were retained for analysis. Along with several questions unrelated to the current research interests, participants completed the following daily measures:

We measured participants’ daily FWB using a single-item, graphical slider scale (“Using the slider provided, please indicate the extent to which you believed you had free will today”). The scale ranged from 0 (*no belief in free will today*) to 100 (*absolute belief in free will today*).

We measured participants’ sense that they controlled their actions and were free to choose that day using single-item, graphical slider scales (“Using the slider provided, please indicate the extent to which you believed you were in control of your actions today”; “…you were free to choose whatever you wanted to do today”). Scores could range from 0 (*no control/no choice at all today*) to 100 (*complete control/complete choice today*). Scores on these two daily measures were averaged to form a composite control/choice variable (within-person reliability = 0.54; see Nezlek, 2017).

As our focal criterion variables, we measured participants’ daily stress (“Today, I felt stressed”) and daily depression (“Today, I felt depressed”) using four-point scales (1 = *not at all*, 4 = *very much*). Depression is an element of the unpleasant affect component of subjective well-being (Diener et al., 1999).

### Results and Discussion

#### Lay Definitions of Free Will

We coded participants’ open-ended responses using Monroe and Malle’s (2010) coding scheme. Specifically, we coded the responses the question “Please explain what you think it means to have free will” in terms of whether participants noted: (a) making decision or choices, (b) doing what they want, and (c) acting without internal or external constraints. Shown in **Table 2**, consistent with Monroe and Malle (2010, 2014), the majority of participants’ definitions of free will referred to the ability to decide/choose, doing what one wants, and/or being free of constraints. During the coding and analysis it was also clear that none of our participants defined free will as reliant upon notions of indeterminism, magical causation or other qualities needed for the type of free will debated by philosophers (see Monroe and Malle, 2010; Baumeister and Monroe, 2014, for discussions).

**Table 2:** Content coding of the folk definitions of free will.

Coding category	Percentage coder agreement	Kappa of agreement	Percentage of participants mentioning the category
Ability to make a decision/choice	91	0.81	64
Doing what you want	84	0.69	50
Acting without constraints	87	0.72	69

*Definitions of coding categories were taken from Monroe and Malle (2010).*

#### Daily Stress and Depression

Given the nested structure of the data (daily responses nested within participants), analyses were performed using multilevel modeling (Nezlek, 2012). Analyses were performed using the lme4 package (Bates et al., 2015) in R, with maximal but uncorrelated random effects (i.e., random slopes and intercepts by participants; including correlations among the random effects led to problems with convergence; Barr et al., 2013). All predictors were person-centered to control for between-person variance in the predictors. We did not model time (days) because we had no theoretical reason to expect time to influence daily changes in stress or depression across the 14-days.

On average participants completed 10.74 (*SD* = 3.75) of the 14 daily surveys (range = 13; total daily surveys completed = 944). **Table 3** shows descriptive statistics and the proportion of variance at the within- and between-person levels for each of the measures we employed.

**Table 3:** Means, standard deviations, and proportion of variance in the predictors and outcome variables at the within- and between-person levels.

Measures	*M*	*SD*			
	
		Between		Within	
Choice/control	75.99	17.06	(60%)	13.84	(40%)
FWB	75.49	20.75	(61%)	16.73	(39%)
Stress	2.28	0.63	(38%)	0.79	(62%)
Depression	1.82	0.64	(44%)	0.72	(56%)

**FIGURE 1:**
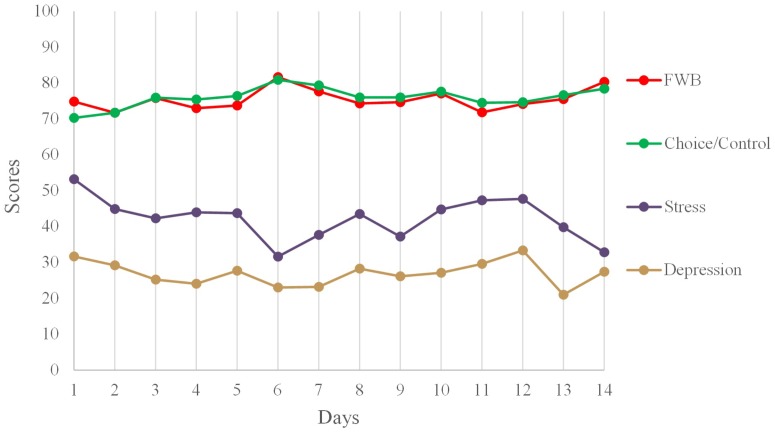
Mean levels of the two main predictor variables (combined choice/control and free will beliefs) and the two criterion variables (stress and depression) across days. Stress and depression have been rescaled (from 1–4 to 0–100) for illustration.

As expected, daily fluctuations in choice/control were significantly associated with daily fluctuations in participants’ FWB, *b* = 0.51, *SE* = 0.07 (95% Wald confidence interval [CI]: 0.38, 0.65; here, FWB was the outcome variable in the analysis). Shown in **Table 4**, both daily FWBs and daily choice/control beliefs significantly predicted daily fluctuations in stress and depression when modeled alone. However, when daily FWBs and daily choice/control were modeled together to predict daily stress and depression, only daily choice/control emerged as a significant predictor. Put differently, at the within-person level, daily changes in FWBs did not account for significant variability in daily stress and depression over and above the contributions of daily changes in choice/control. **Figure 1** shows the means of FWB, choice/control, stress, and depression across the 14 days.

**Table 4:** Linear mixed effects models predicting daily stress and daily depression from daily FWB and daily choice/control (alone and with both simultaneously).

	Daily FWB			Daily choice/control		
	*b*	*SE*	Wald 95% CI	*b*	*SE*	Wald 95% CI
**Daily stress**						
FWB alone	**-0.007^∗^**	0.002	[**-**0.012, **-**0.002]	–	–	–
Choice alone	–	–	–	**-0.010^∗^**	0.003	[**-**0.015, **-**0.004]
FWB and choice	**-**0.002	0.002	[**-**0.006, 0.002]	**-0.009^∗^**	0.003	[**-**0.014, **-**0.003]
**Daily depression**						
FWB alone	**-0.008^∗^**	0.003	[**-**0.013, **-**0.003]	–	–	–
Choice alone	–	–	–	**-0.011^∗^**	0.003	[**-**0.017, **-**0.007]
FWB and choice	**-**0.002	**-**0.002	[**-**0.007, 0.002]	**-0.01^∗^**	0.003	[**-**0.016, **-**0.005]

*FWB, free will belief. ^∗^p < 0.05 (based on the Wald 95% confidence interval not containing zero).*

Like in Study 1, we tested whether there was a significant decrease in the regression weight for daily FWB when modeled with daily choice/control to predict each of daily stress and daily depression compared to when FWB was modeled alone. We used Rockwood and Hayes’s (2017; see Zhang et al., 2009) MLmed SPSS macro for multilevel mediation to perform these analyses (with 10,000 Monte Carlo samples). Analyses showed that the relations between daily FWB and daily stress (within-subject indirect relationship = -0.004, *Z* = -2.77, *p* = 0.006, 95% Monte Carlo CI: -0.007, -0.001) and daily FWB and daily depression (within-subject indirect relationship = -0.005, *Z* = -3.38, *p* = 0.001, 95% Monte Carlo CI: -0.008, -0.002) were significantly reduced from their zero-order relationships when statistically controlling for a sense of personal control.

These findings are consistent with our trait-level findings reported in Study 1: associations between participants’ subjective well-being (in this case, daily stress and depression) and FWBs appear to be due to the co-variation between FWB and beliefs about having control and being able to choose.

## General Discussion

Across two studies we investigated the role of personal control and choice in the relationship between FWB and subjective well-being. Previous research has provided evidence for the predictive value of FWB on such outcomes (e.g., Crescioni et al., 2015). Here, we show that this association can be explained by perceived control/choice. Study 1 showed that trait-level belief in personal control significantly uniquely predicted SWL and stress, whereas FWB did not. Study 2 confirmed that within-person daily fluctuations in stress and depression are not significantly predicted by daily changes in FWB over and above the contribution of personal control/choice.

Previous research has shown that the association between FWB and judgments of others’ morality/blame is due to perceived capacity for choice (Monroe et al., 2017). The current studies extend this by showing that like judgments of others’ behavior, the relationship between FWB and personal life outcomes, relevant to subjective well-being, is also due to co-variation with control/choice. Crescioni et al. (2015) showed that although both self-efficacy and locus of control were correlated with FWB, they did not explain the association between FWB and life outcomes (meaning in life and SWL). We chose to focus on measures of control/choice that more closely reflected the nature of layperson conceptions of free will (Monroe and Malle, 2010). Unlike Monroe et al. (2017), who manipulated/measured choice using vignettes, we used a self-report measure of the degree to which participants believed in the ability to control their behavior or have the capacity for choice. These measures effectively captured the key elements of the lay concepts of free will to the extent that they reduced the predictive utility of FWB on perceived stress and depression.

Much recent research has investigated the role of FWBs in a number of life outcomes, as well as psychological well-being. Here, we provide evidence for the role of personal control/choice in explaining why FWB predicts stress and depression. For laypeople, belief in free will fundamentally means having the capacity to make choices and control one’s life (Monroe and Malle, 2010), and our Study 2 findings of participants’ definitions of free will confirm this. This perception of personal control appears to be protective of perceived stress and depression such that individuals with strong belief in the degree to which they control their lives may be less likely to negatively react to stressful life events. We also provide further evidence for the role of perceived control in stress and depression. This goes beyond previous research, by utilizing measures of control/choice that are closely aligned to high level beliefs in free will. Future research should investigate the relative power of these different aspects of choice in predicting stress and depression.

Although we show that the predictive utility of FWB on personal life outcomes is abolished when controlling for personal choice, it remains possible that FWB does have unique predictive utility in other contexts. Indeed, the modest correlation between FWB and personal control suggests that FWB and personal control are not precisely the same thing. Nonetheless, recent work (Monroe et al., 2017) shows that the relationship between FBW and morality is similarly explained by notions of personal control. As such future research should seek to determine which behaviors or outcomes might be predicted by FWB over and above personal control.

Further research should also attempt to identify the direction of these relationships. For instance, much research on FWB assumes that belief or disbelief in free will drives life outcomes and personal well-being. However, while control beliefs influence how someone copes with a stressful event, this coping also feeds back into the individual’s sense of personal control (Anderson, 1977). As such, while belief in free will/choice may be protective of subjective well-being, stressful life events may also lead to a reduction in a sense of personal control.

## Ethics Statement

Ethical approval was granted by the University of Essex Faculty of Science and Health Ethics Sub-Committee (Approval Nos. PG1602 and PG1603). Prior to testing, all participants were informed of their right to withdraw from the research at any time. All participants signaled their consent to participate.

## Author Contributions

The research was conducted online and analyzed by PG and MC. All three authors conceived of and designed the studies, contributed to writing the manuscript, and approved the final version.

## Conflict of Interest Statement

The authors declare that the research was conducted in the absence of any commercial or financial relationships that could be construed as a potential conflict of interest.
